# Genome wide expression analysis in HPV16 Cervical Cancer: identification of altered metabolic pathways

**DOI:** 10.1186/1750-9378-2-16

**Published:** 2007-09-06

**Authors:** Carlos Pérez-Plasencia, Guelaguetza Vázquez-Ortiz, Ricardo López-Romero, Patricia Piña-Sanchez, José Moreno, Mauricio Salcedo

**Affiliations:** 1 Unidad de Investigación Biomédica en Cáncer, Instituto de Investigaciones Biomédicas, Universidad Nacional Autonóma de Mexico (UNAM), (INCAN), Mexico City, Mexico; 2 Laboratorio de Oncología Genómica, Unidad de Investigación Médica en Enfermedades Oncológicas, Hospital de Oncología, CMN Siglo XXI-IMSS, Mexico; 3 Unidad de Investigación Médica en Enfermedades Autoinmunes, Hospital de Especialidades, CMN Siglo XXI-IMSS, Mexico

## Abstract

**Background:**

Cervical carcinoma (CC) is a leading cause of death among women worldwide. Human papilloma virus (HPV) is a major etiological factor in CC and HPV 16 is the more frequent viral type present. Our aim was to characterize metabolic pathways altered in HPV 16 tumor samples by means of transcriptome wide analysis and bioinformatics tools for visualizing expression data in the context of KEGG biological pathways.

**Results:**

We found 2,067 genes significantly up or down-modulated (at least 2-fold) in tumor clinical samples compared to normal tissues, representing ~3.7% of analyzed genes. Cervical carcinoma was associated with an important up-regulation of Wnt signaling pathway, which was validated by in situ hybridization in clinical samples. Other up-regulated pathways were those of calcium signaling and MAPK signaling, as well as cell cycle-related genes. There was down-regulation of focal adhesion, TGF-β signaling, among other metabolic pathways.

**Conclusion:**

This analysis of HPV 16 tumors transcriptome could be useful for the identification of genes and molecular pathways involved in the pathogenesis of cervical carcinoma. Understanding the possible role of these proteins in the pathogenesis of CC deserves further studies.

## Background

Cervical carcinoma (CC) is one of the most common cancers and a leading cause of death among women worldwide. Epidemiologic and experimental studies have identified a causal role of high risk HPV types in cervical carcinogenesis [1,2]. HPV 16 is the predominant type with prevalence ranging from 43.9% to 72.4%, followed by HPV 18 with 15% of the CC cases [3]. Persistent viral infection in combination with strong, constitutive expression of E6 and E7 viral oncogenes is a necessary step for malignant transformation because these proteins interact with p53 and pRB proteins leading to their degradation and deregulation of the cell cycle [4,5]. Moreover, E6 and E7 target additional cellular proteins and transcriptional regulators [reviewed in [6,7]], cellular targets of E6 and E7 includes several cellular transcription regulators [8,9]. Finally, at DNA level E6 and E7 deregulate cell proliferation and induce genetic instability, which promote the accumulation of mutations and aneuploidy [10]. In conclusion, viral oncoproteins have a profound impact in global profile of expressed genes, which could be analyzed by transcriptome wide analysis methodologies. One of these techniques is DNA oligonucleotide-based microarray technology, which allows a rapid and high throughput detection of thousands of transcripts simultaneously [11-13].

There are several studies published related to gene expression profiles in HPV infected cells, which have mainly addressed gene expression levels in cultured keratinocytes [14-19]; these studies explain several aspects concerned to molecular mechanisms induced or modified by E6 and E7 viral oncoproteins. Regarding changes in gene expression in CC clinical samples, there are a few papers comparing normal cervical expressed genes versus tumor samples [20-23], with the major aim to find potential tumour markers of clinical value none of them has examined whole genome expression. Hence, to further identify genes and molecular pathways associated with HPV16-CC, we performed a whole genome expression profile of primary tumors and normal cervical tissues.

Our results identified novel deregulated genes and candidate regulatory pathways in CC tissues. The altered regulatory pathways identified were: focal adhesion MAPK, calcium signaling and Wnt. This last one is involved in various differentiation events during embryonic development in mammals leading to tumor formation when is aberrantly activated [24,25]. Overexpression of four genes of the Wnt signaling pathway was validated by *in situ *hybridization on Tissue Microarrays (TMAs). These results improve our knowledge about molecular alterations in CC pathogenesis.

## Results

### Gene expression profile of HPV 16 squamous cervical carcinoma

Our major aim was to identify the gene expression profile in HPV 16 positive CC by wide genome analysis. Therefore, we used the Human Whole Genome CodeLink microarrays, which have the enough sensitivity to detect as little as one transcript per cell and the specificity to distinguish among highly homologous sequences. This array contains a broad range of genes derived from publicly available mRNA sequences (over 55,000 genes and ESTs probes) [26]. After cluster filtering was done (see Methods), 6,007 probes corresponding to 3,248 characterized transcripts were obtained. We reanalyzed this data set using a supervised approach with Significance Analysis of Microarrays (SAM) [27]; obtaining 1,007 and 1,060 genes that were significantly up and down-regulated, respectively with a false discovery rate <10% (Additional file 1).

### Cluster Analysis

**Figure 1:**
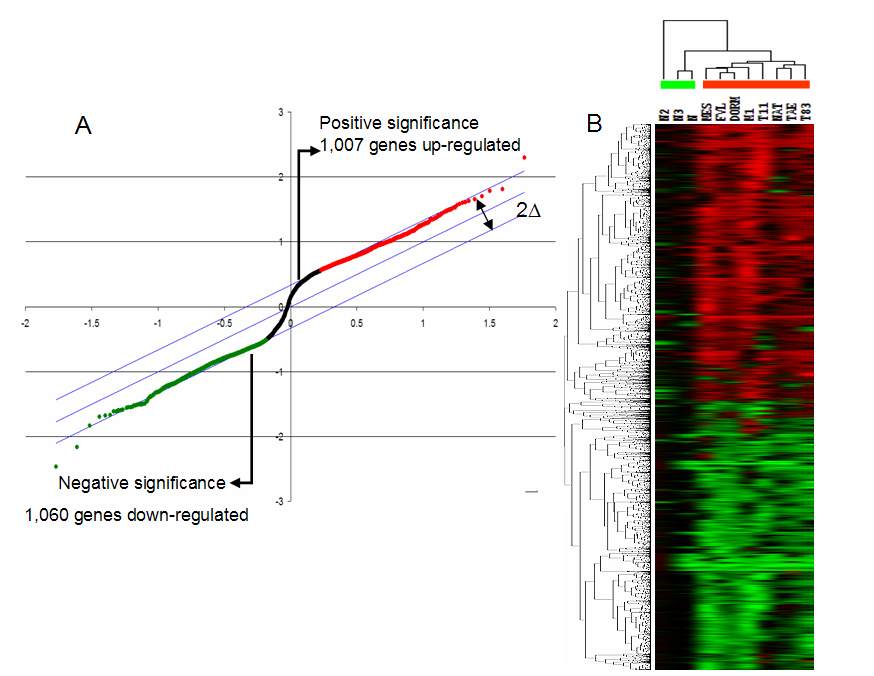
**Gene expression profile in cervical carcinoma HPV 16+ as identified by significance analysis of microarrays (SAM) and Eisen Cluster analysis**. **A. **SAM plot representing modulated genes. The delta was set to support a false discovery rate of 10% or less. Genes with expression levels that are statistically beyond delta in either direction are plotted either above (induced, red) or below (repressed, green) the control group. Genes which expression level did not change more than the set delta in either direction were considered to be not statistically significantly different at the set false discovery rate. **B: **Cluster analysis of cDNA microarray data. Microarray data were analyzed by the Eisen Hierarchical Cluster program and visualized with TreeView. The cluster shows 2,836 genes. Each row represents a gene, whereas each column corresponds to a tissue sample, the color line below tissue samples indicates, a) green: normal samples and b) red: tumor samples. The relative abundance of each gene in the tissue correlates with color intensity (red induced; green, repressed; black, no change). On the dendogram, all eight invasive cervical cancers clustered together such as two of the tree normal samples (N and N1) cluster indicating their similarity based on expression profile.

To obtain a graphical representation of the differences between normal and tumor tissues, cluster analysis was performed on all samples. The phylogenetic tree resulting from the hierarchical complete linkage clustering algorithm is shown in figure 1b. In this method of clustering, relationships among objects (genes or samples) are represented by a tree whose branch lengths reflect the degree of similarity between the objects, as assessed by a pairwise similarity function. The computed trees can be used to arrange individual samples in the original data table, this allows the samples or groups of samples with similar expression patterns to be shown adjacent to each other. In general, tumor samples showed a higher grade of homology among them compared with normal samples which had a more heterogeneous gene expression profile. This observation suggests that although HPV infection is associated with several changes in the host cell, in late stages of the carcinogenesis process the pattern of gene expression induced by HPV 16 infection is very similar. However, it is also possible that the similarities observed in tumor samples were due to the rigorous procedure used to select our patients that included age, clinical stage, and contraceptive oral status.

### Pathway Analysis

**Figure 2:**
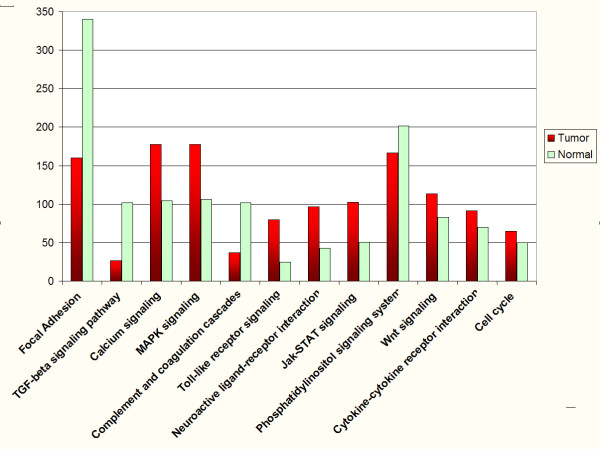
KEGG-based metabolic pathways identified to be altered in cervical carcinomas. To know cellular pathways involved and the extent of alteration in CC, we imported a list of significantly up and down-regulated genes to Pathway Express. Then the Impact Factor obtained was graphed.

**Figure 3:**
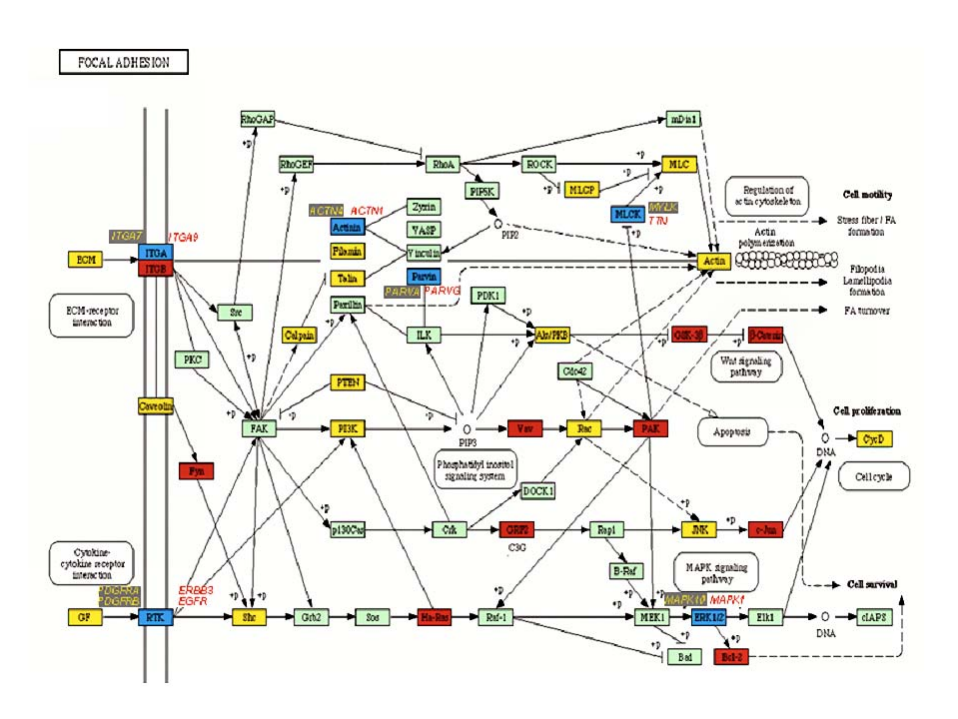
KEGG-Cellular Pathways integrating our expression data of Focal Adhesion. Yellow boxes indicates a down regulation of expression in tumor cervices samples. Red boxes indicates a higher gene expression in cervical carcinoma samples. Blue boxes indicates that a member of indicated gene family is expressed in normal tissues, and other member in tumors. Green boxes indicate that the gene was not altered.

**Figure 4:**
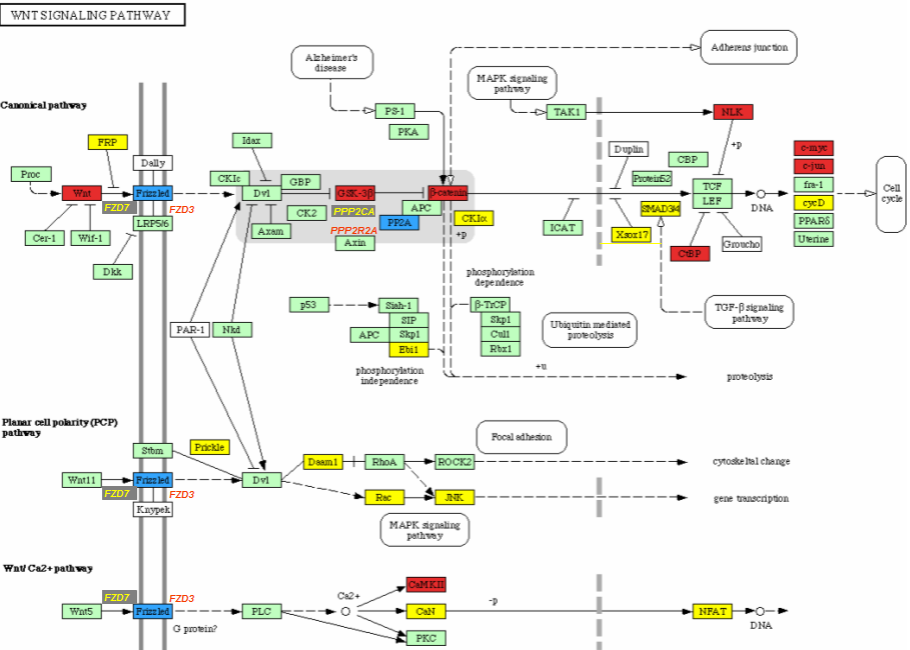
In situ hybridization of cervical tissues. Capital letters show *In situ *hybridizations for FZD2, GSK3B, C-MYC and PPARD probes in Normal HGSIL and CC samples.

In order to identify the biological meaning of changes in gene expression, genes examined by significance analysis of microarrays (SAM) were submitted to the visualization tool Pathway Express (PE, a component of Onto-Tools suite). This recently developed bioinformatic tool is employed for visualizing expression data in the context of KEGG biological pathways, the importance PE is that retrieves an impact factor (IF) of the entire pathway involved, which can help to obtain a clearer notion of the alteration level in each biological pathway [28]. We imported a list of significantly up and down regulated genes into the program to convert the expression data into illustrations in an attempt to explore altered mechanisms in CC (additional file 1). To overcome any possible incorrect IF in altered pathways due to different size of samples, we submitted a similar quantity of up and down regulated genes onto PE. This allowed confirming that genes involved in several metabolic pathways were altered in HPV16+ CC (Figure 2). As seen, cellular pathways altered in CC are related to the maintenance of malignant process such as decrease in focal adhesion, overexpression in Wnt-signalling and cell cycle pathways (Figures 2, 3 and 4).

One of the most altered cellular pathways in CC was focal adhesion. We were able to identify a reduced expression of many of genes of this pathway, especially those coding for extracellular matrix in tumor samples. Nevertheless, some of them, such as laminins were slightly up-regulated (Additional file 2).

In this work we analyzed squamous CC with clinical stage IIB (according to FIGO nomenclature); this kind of tumours are highly invasive, hence the down modulation of some genes related to focal adhesion is an important step that could precede the highly metastatic grade in more advanced clinical stages IIIA and IIIB.

An important gene linked to this process is *PTEN *(Figure 3), which is considered to be a candidate tumour suppressor gene and has been frequently found mutated in several types of human tumors including cervical carcinomas [29]. Concordantly, this gene was down regulated in CC (Additional file 2).

Of the up-regulated pathways found, we focused on the Wnt signalling pathway. A connection between Wnt signaling and human disease has been established by findings in malignancies such as colorectal [30], gastric [31], CC [32] and hepatocellular carcinomas [33]. The common mechanism altered in those examples is the activation of gene transcription by an accumulation of β-catenin in the nucleus, with recent evidence of an aberrant nuclear and cytoplasmic expression of β-catenin in CC cells [34], indicating a possible role in cervical carcinoma development. Genes of this pathway up-regulated in CC tumor cells were JUN, MYC, FZD2, RAC1, GSK3B and CTNNB1, and a few others (Additional file 2). Because of the possible relevance of this pathway, we validated these findings by in situ hybridization of four of its genes.

### In situ hybridization (ISH)

We selected four genes (FZD2, GSK3β, PPARδ and C-MYC) of the Wnt signaling pathway to validate the over-expression found in the microarray assay in CC samples.

**Figure 5:**
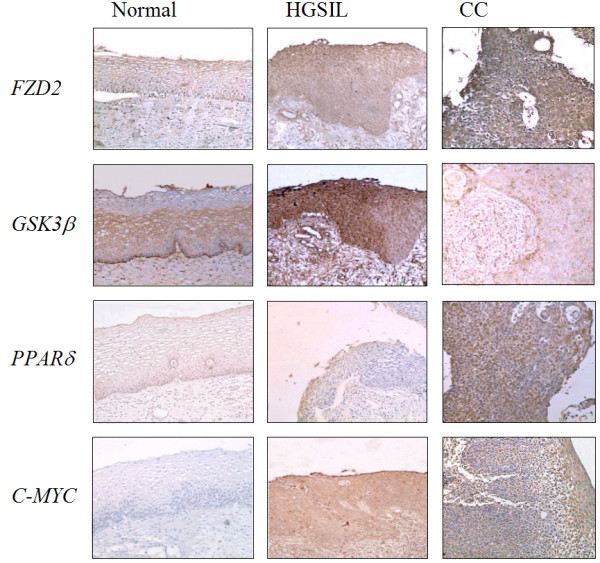
KEGG-Cellular Pathways integrating our expression data of WNT-signalling pathways. Yellow boxes indicates a down regulation of expression in tumor cervices samples. Red boxes indicates a higher gene expression in cervical carcinoma samples. Blue boxes indicates that a member of indicated gene family is expressed in normal tissues, and other member in tumors. Green boxes indicate that the gene was not altered.

Normal cervical tissues showed a dull staining for FZD2, C-MYC and PPARδ in majority of the cells and layers of the epithelium, whereas GSK3β stained bright and strong in the cytoplasm in the middle layers of the epithelium. The staining was also observed in the adjacent stroma. All high grade precancerous cervical lesions (HGSIL) showed numerous FZD2, GSK3β, C-MYC and PPARδ positive cells. The percentage of FZD2, GSK3β, C-MYC and PPARδ positive cells, was higher in HGSIL than in normal cervical epithelium. Even a strong staining was observed in the adjacent stroma compared to the reaction present in normal cervical epithelium surrounding stroma. In general, the intensity of reaction and the number of FZD2, GSK3β, C-MYC and PPARδ positive cells in CC were slightly but stronger than HGSIL and normal samples (Figure 5).

## Discussion

In the present work, we identified genes that were up-regulated in HPV16 CC by means of whole genome microarrays in relation to normal cervical HPV negative cells. Although we did not compare different tissue samples infected with distinct HPV types, the gene list and cellular pathways reported here could reflect not only alterations induced by this viral type but also with other high risk HPVs. In this regard, some of genes mentioned here have been previously reported as typically altered in CC or involved in the process of carcinogenesis. These include c-myc, ras, fos, NF-κB, EGFR, β-catenin, etc. [35-38] (Additional file 2). Strong evidence in the literature indicates a determinant role of these genes in cervical carcinogenesis and this validates the approach employed here.

The protein network in KEGG is an abstract graphical representation of gene products, that include not just the pathway resulting from direct protein-protein interactions, but also the metabolic network viewed as a framework of enzymes and the regulatory gene network viewed at the level of interaction between transcription factors and target products. The "PATHWAY" database is a collection of manually drawn diagrams called the KEGG reference pathway diagrams (maps), each corresponding to a known network of functional significance [39].

Thus, a systematic evaluation of differential regulation in metabolic pathways is shown in Figure 2. Some of these biological pathways have an important role in the carcinogenesis process in several models meaning that could have it also in CC. In this context, Wnt pathway has been proposed as a promoter of HPV-induced human keratinocyte transformation, also promotes activation of the cell cycle, following the initiation of frizzled cascade, a rise in the level and accumulation of β-catenin in the nucleus induces transcriptionally active products of the Tcf family of transcription factors, which target positive regulators of cell cycle, such as c-myc, cyclin D and c-jun [40,41] all of them over-expressed in our analysis.

**Figure 6:**
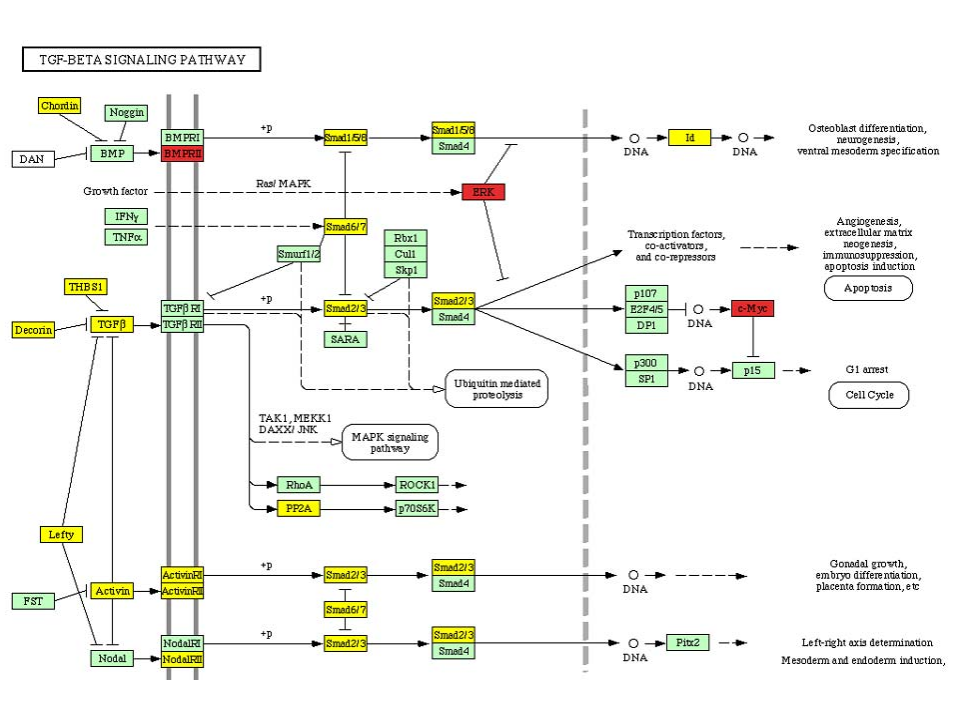
KEGG-Cellular Pathways integrating our expression data of TGF-beta signalling pathways respectively. Yellow boxes indicates a down regulation of expression in tumor cervices samples. Red boxes indicates a higher gene expression in cervical carcinoma samples. Blue boxes indicates that a member of indicated gene family is expressed in normal tissues, and other member in tumors. Green boxes indicate that the gene was not altered.

As seen in (figures 2 and 6), TGF-β pathway was down regulated in CC. This pathway is critical for maintaining homeostatic of growth control not only in premalignant cells but also in cells progressing through the early stages of carcinogenesis. Central to its growth inhibitory effects, at least *in vitro*, is its ability to suppress expression of c-Myc and to enhance expression of the cyclin-dependent kinase inhibitors p15INK4b and p21CIP1. Effects of TGF-β on genomic stability, replicative senescence, and apoptosis also come into play in particular cell types [reviewed in [42]]. Consistently, in the present analysis we found a down regulation in TGF-β signalling pathway in cervical tumours. A previous report by Nees et al. has shown a TGF-β strong down modulation by HPV-16 E6 and E7 viral oncoproteins. They also demonstrated that many of the genes that were perturbed by E6 and E7 in differentiating keratinocytes were TGF-β-responsive [43].

Smad proteins play a key role in the intracellular signaling of TGF-β. Upon TGF-β receptor activation, Smad2 and Smad3 become phosphorylated and form heteromeric complexes with Smad4. These complexes translocate to the nucleus where they control expression of target genes. Lee *et al *have shown that HPV-16 E7 oncoprotein blocks TGF-β signaling by blocking binding of the Smad complex to its target DNA sequence. E7 binds to Smad2, Smad3, and Smad4, and blocks binding of Smad3 to its DNA binding site [44]. Moreover, at transcriptional level loss of Smad 2 and 4 expressions in human cervical carcinomas has been observed [45]. We showed in the present analysis a down regulation of Smad's expression in cervical tumours (Additional file 2). These observations could support the function of TGF-β as tumor suppressors in cervical epithelia and also confirm that loss of TGF-β expression in HPV-infected keratinocytes is not restricted to cell cultures. Thus, down-regulation of TGF-β and its signaling pathway by HPV could represent an important mechanism contributing to growth stimulation, immortalization, and carcinogenesis.

**Figure 7:**
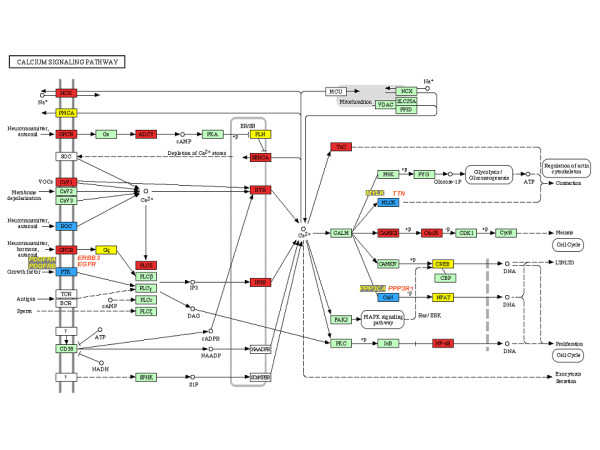
KEGG-Cellular Pathways integrating our expression data of Calcium signalling pathways respectively. Yellow boxes indicates a down regulation of expression in tumor cervices samples. Red boxes indicates a higher gene expression in cervical carcinoma samples. Blue boxes indicates that a member of indicated gene family is expressed in normal tissues, and other member in tumors. Green boxes indicate that the gene was not altered.

Calcium (Ca^2+^) is a universal intracellular second messenger that activates many cellular functions, such as gene expression, proliferation, differentiation, apoptosis and aging, cell adhesion, cell migration, etc. An increase in cytosolic calcium concentration has been related to oncogene activation [46], wound repair as well as in the initiation and promotion of tumors [47]. A key element of calcium-dependent cell activation is the release of calcium ions into the cytosol from the lumen of the endoplasmic reticulum (ER) through calcium channels activated by the second messenger myo-D-inositol-1,4,5-trisphosphate (IP3R) and ryanodine (RyR)-dependent Ca2+ channel [48]. In the present work, we found up-regulation of both calcium channels suggesting an increase of Ca^2+ ^in CC cells (Additional file 2 and figure 7) [49]. IP3R overexpression in cultures of gastric cancer cells correlated with peritoneal dissemination [50]. Meanwhile activation of ryanodine receptors was shown in a neuroblastoma cell line [51]. Although we found co expression of SERCA genes, which are involved in decrease levels of cytosolic calcium [reviewed in [47]], there was also an-up regulation of ryanodine and IP3 receptors; which are involved in raising calcium levels. Therefore, regulation of Ca^2+ ^levels in cancer cells appear to be more complex and subsequent studies are needed to clarify it.

## Conclusion

Previous expression profiling studies have been limited to the identification of genes aberrantly expressed in CC that could be used as molecular markers. We combined wide transcriptome analysis and biological pathway characterization to identify cellular pathways with an aberrant expression of a significant proportion of genes in CC. This allowed us to confirm aberrations in molecular pathways known to be important in cervical cancer such as Wnt and TGF-β signaling, and cell cycle regulation with validation of over-expression of genes of the Wnt-signalling pathway. The importance of this work is that it defines new altered metabolic pathways, such as calcium signaling, and genes involved improving our knowledge of the molecular alterations in CC pathogenesis. The meaning of the role of these proteins in the pathogenesis of CC deserves further studies.

## Methods

### Tissues for array hybridization

Eight fresh biopsies of Invasive squamous CC Stage IIB HPV16, were taken during colposcopy. Three normal cervixes from women who had been subjected to hysterectomy due to uterine myomatosis were obtained; these were also verified to be negative with Pap smears and also confirmed by histopathological analysis. All samples were taken from the Dysplasia Clinic at General Hospital of Mexico, SS and all patients were in reproductive age and none of them had received hormonal therapy or contraceptives. The described procedures were evaluated and approved by the local ethics committee of the Mexican Institute of Social Security and written informed consent was obtained from patients.

All tissue samples were longitudinally divided in three sections, the central part was snapped frozen in liquid nitrogen and stored at -70°C until nucleic acid extraction, and the other two parts were fixed overnight in 70% ethanol and paraffin embedded at the Department of Pathology, Oncology Hospital, National Medical Center SXXI, Mexico. Serial sections from these fractions were stained by Haematoxilin/Eosin and inspected to confirm the presence of at least 70% of tumoral cells in the tissue. Multiple step sections were made and classified according to the International Federation of Obstetrics and Gynecology (FIGO). Normal tissue samples were considered as such when at least 80% of the sample consisted of epithelial ectocervical normal tissue and HPV infection absence.

### Tissue samples for ISH

Fifteen LSILS paraffin blocks were used to obtain the normal cervical tissue samples which were adjacent to the low grade lesions, and also 50 paraffin blocks: 25 CC and 25 HGSIL were randomly collected from the files of the National Center of Dysplasias in Mexico City. The tissue microarrays were constructed as previously described [52]. One of the TMAs contained the 25 HGSIL tissues, another one contained all the CC samples and finally a "normal tissue microarray" was elaborated.

### HPV detection and typing

Genomic DNA was extracted from the phenol phase left by TRIzol reagent (Gibco BRL, USA) and amplified by PCR specific primers for HPV 16 [53]. PCR products were separated by electrophoresis on a 1% agarose gel. Only dysplastic lesions with HPV 16 infection were included in this study. PCR was performed using a Perkin Elmer Thermal cycler 480, with the following conditions, after an initial 5-min denaturing step at 95°C, the following 35 cycles were at 95°C for 30 sec, 55°C for 30 sec, and 72°C for 1 min.; followed by a single step of 72°C for 5 min. PCR mix without DNA was used as negative control.

### *In Situ *Hybridization (ISH)

Briefly, five-micron tissue sections were obtained from TMA paraffin blocks, deparaffinized and rehydrated in a graded ethanol series and transferred to PBS solution. Tissues were digested with DNase solution (1 μg/ml) for 10 min at 37°C. Endogenous peroxidase was inactivated by incubation in hydrogen peroxide in methanol for 3%. Sense and antisense probes for FZD2, GSK3β, PPARδ and c-myc genes were generated by single-strand PCR using specific cDNA obtained from SiHa cells RNA as template and labeled with biotin-16-dUTP (Roche). Hybridization was carried out for 14 hrs at 37°C. After hybridization, stringent wash in Tris-buffered saline with Tween buffer 1× (TBST 10×: 500 nmol/L TrisHCL, pH 7.6, 3 mol/L NaCl, 1% Tween 20), at 55°C for 20 min was done. Finally the signal was developed using GenPoint System (DAKO, Glonstrup Denmark) according to manufacturer. Finally, the slides were hematoxylin counterstained, dehydrated in graded ethanol and mounted. Negative controls for ISH were carried out with the sense probes or with a treatment with RNAse solution (100 mg/ml for 30 min at 37°C) prior ISH.

### Interpretation and quantification

Cells were noted as positive for FZD2 when the staining was confined to the plasma membrane, while GSK3β, C-MYC and PPARδ were expressed in the cytoplasm. Only the neoplastic region or the epithelium of each tissue section was evaluated. Two slides were analyzed for each sample. To asses the stained cytoplasm the slides were reviewed at ×40 magnification. The percentage of positive cells in each tissue section was estimated based on a semiquantitative scale where: 0% (absent), 1–5% (sporadic), 6–25% (local), 26–50% (occasional), 51–75% (majority), 76–100% (large majority of the tumoral area or epithelium). After the examination of the slides by three independent observers, a global agreement was reached. Regarding staining, only those with an intensity of: + (dull), ++ (clear) or +++ (bright) were scored.

## Competing interests

The author(s) declare that they have no competing interests.

## Authors' contributions

C.P.P. carried out microarray experiments, the bioinformatics analysis, writing the manuscript and conceptualized the study. GVO carried out the ISH experiments and statistical analysis RLR participated in discussions. PPS participated in the bioinformatic analysis and database comparisons. JM participated in critical review and writing of the manuscript. MS is the principal investigator and was involved in the conceptualization, design discussion and writing of the manuscript. All authors read and approved the final manuscript.

## Supplementary Material

Additional file 1 Genes with a significantly altered gene expression in CC. Click here for file

Additional file 2 KEGG-pathways and genes altered in cervical tumours. Table containing KEGG pathways and related genes which were significantly up or down expressed in cervical carcinoma. Click here for file
