# Supplementary material for: Genome wide expression analysis in HPV16 Cervical Cancer: identification of altered metabolic pathways
Source: Infect Agent Cancer. 2007 Sep 6;2:16. doi: 10.1186/1750-9378-2-16 (PMC2034543; doi:10.1186/1750-9378-2-16)
Supplement: Additional file 2 — KEGG-pathways and genes altered in cervical tumours. Table containing KEGG pathways and related genes which were significantly up or down expressed in cervical carcinoma. [file 1750-9378-2-16-S2.doc]

Table 1. KEGG-pathways and genes altered in cervical tumours.

| **Down regulated** | | | **Over-expressed** | | | | |
| --- | --- | --- | --- | --- | --- | --- | --- |
| Gene | Gene Name | Ratio^a^ | Gene | | Gene Name | Ratio^a^ | |
| **FOCAL ADHESION** | |  | **FOCAL ADHESION** | | |  | |
| MYLK | myosin, light polypeptide kinase | -6.2 | JUN | v-jun sarcoma virus 17 oncogene | | 4.8 | |
| IGF1 | insulin-like growth factor 1 (somatomedin C) | -5.2 | TTN | Titin | | 3.9 | |
| COL1A2 | collagen, type I, alpha 2 | -5.1 | RAC1 | ras-related C3 botulinum toxin substrate 1 (rho family, small GTP) | | 3.2 | |
| COL3A1 | collagen, type III, alpha 1 | -4.8 | BCL2 | B-cell CLL/lymphoma 2 | | 3.1 | |
| COL4A6 | collagen, type IV, alpha 6 | -4.2 | MAPK1 | mitogen-activated protein kinase 1 | | 3.1 | |
| MYL6 | myosin, light polypeptide 6. | -4.1 | RAPGEF1 | Rap guanine nucleotide exchange factor (GEF) 1 | | 3.1 | |
| ACTG1 | Actin, gamma 1 | -4.1 | VAV1 | vav 1 oncogene | | 2.9 | |
| COL6A2 | collagen, type VI, alpha 2 | -4 | ACTN1 | actinin, alpha 1 | | 2.9 | |
| THBS1 | thrombospondin 1 | -3.9 | GSK3B | glycogen synthase kinase 3 beta | | 2.8 | |
| COL5A1 | collagen, type V, alpha 1 | -3.9 | MAPK10 | mitogen-activated protein kinase 10 | | 2.7 | |
| ACTB | Actin, beta | -3.8 | PARVG | parvin, gamma | | 2.5 | |
| COL4A1 | collagen, type IV, alpha 1 | -3.8 | LAMA4 | laminin, alpha 4 | | 2 | |
| ACTC | Actin, alpha, cardiac muscle | -3.7 | RELN | reelin | | 1.9 | |
| LAMB2 | laminin, beta 2 (laminin S) | -3.7 | LAMC2 | laminin, gamma 2 | | 1.9 | |
| FLNC | filamin C, gamma (actin binding protein 280) | -3.5 | PAK3 | p21 (CDKN1A)-activated kinase 3 | | 1.9 | |
| COL6A1 | collagen, type VI, alpha 1 | -3.5 | FYN | FYN oncogene related to SRC, FGR, YES | | 1.7 | |
| LAMA2 | laminin, alpha 2 (merosin, congenital muscular dystrophy) | -3.4 | CTNNB1 | catenin (cadherin-associated protein), beta 1, 88kDa | | 1.7 | |
| PIK3R1 | phosphoinositide-3-kinase, regulatory subunit 1 (p85 alpha) | -3.4 | ITGA9 | integrin, alpha 9 | | 1.5 | |
| ACTN4 | actinin, alpha 4 | -3.3 | **TGF-BETA** | | |  | |
| PDGFRB | platelet-derived growth factor receptor, beta polypeptide | -3.3 | MYC | v-myc myelocytomatosis viral oncogene homolog (avian) | | 3.7 | |
| TNXB | tenascin XB | -3.3 | MAPK1 | mitogen-activated protein kinase 1 | | 3.1 | |
| CAV2 | caveolin 2 | -3.3 | BMPR2 | bone morphogenetic protein receptor, type II | | 2.9 | |
| PPP1R12A | protein phosphatase 1, regulatory (inhibitor) subunit 12A | -3 | **CALCIUM SIGNALLING** | | |  | |
| SHC1 | SHC (Src homology 2 domain containing) transforming protein 1 | -3 | CACNA1C | calcium channel, voltage-dependent, L type, alpha 1C subunit | | 4.8 | |
| CCND2 | cyclin D2 | -2.9 | RYR2 | ryanodine receptor 2 (cardiac) | | 4.6 | |
| PTEN | phosphatase and tensin homolog (mutated in multiple advanced cancers | -2.8 | SLC8A1 | solute carrier family 8 (sodium/calcium exchanger), member 1 | | 4.5 | |
| TNC | tenascin C (hexabrachion) | -2.7 | TTN | Titin | | 3.9 | |
| PDGFRA | platelet-derived growth factor receptor, alpha polypeptide | -2.7 | ADCY1 | adenylate cyclase 1 (brain) | | 3.7 | |
| AKT3 | v-akt murine thymoma viral oncogene homolog 3 (protein kinase B, gamma) | -2.7 | TNNC1 | troponin C type 1 (slow) | | 3.4 | |
| LAMB1 | laminin, beta 1 | -2.6 | ATP2A1 | ATPase, Ca++ transporting, cardiac muscle, fast twitch 1 | | 3.3 | |
| ITGA7 | integrin, alpha 7 | -2.6 | ERBB3 | v-erb-b2 erythroblastic leukemia viral oncogene homolog 3 (avian) | | 3.3 | |
| EGFR | epidermal growth factor receptor (erythroblastic leukemia viral | -2.6 | GRIN2D | glutamate receptor, ionotropic, N-methyl D-aspartate 2D | | 3.1 | |
| TLN2 | Talin 2 | -2.6 | ITPR1 | inositol 1,4,5-triphosphate receptor, type 1 | | 3.1 | |
| PARVA | parvin, alpha | -2.4 | GRIN1 | glutamate receptor, ionotropic, N-methyl D-aspartate 1 | | 2.8 | |
| FIGF | c-fos induced growth factor (vascular endothelial growth factor D) | -2.3 | CAMK2G | calcium/calmodulin-dependent protein kinase (CaM kinase) II gamma | | 2.3 | |
| **TGF-β** |  |  | CHRM5 | cholinergic receptor, muscarinic 5 | | 2.2 | |
| DCN | decorin | -5.9 | CCKBR | cholecystokinin B receptor | | 2.2 | |
| ID3 | inhibitor of DNA binding 3, dominant negative helix-loop-helix | -4.7 | CAMK4 | calcium/calmodulin-dependent protein kinase IV | | 2.2 | |
| GDF6 | growth differentiation factor 6 | -4.7 | CAMK2D | calcium/calmodulin-dependent protein kinase (CaM kinase) II delta | | 2.1 | |
| THBS1 | thrombospondin 1 | -3.9 | PLCD1 | phospholipase C, delta 1 | | 2.1 | |
| ID2 | inhibitor of DNA binding 2, dominant negative helix-loop-helix | -3.7 | GRM5 | glutamate receptor, metabotropic 5 | | 1.6 | |
| ID1 | inhibitor of DNA binding 1, dominant negative helix-loop-helix | -3.6 | **MAPK SIGNALING** | | |  | |
| LTBP1 | latent transforming growth factor beta binding protein 1 | -3.5 | RASGRP3 | RAS guanyl releasing protein 3 (calcium and DAG-regulated) | | 8.5 | |
| CHRD | chordin | -3.2 | FOS | v-fos FBJ murine osteosarcoma viral oncogene homolog | | 7 | |
| ACVRL1 | activin A receptor type II-like 1 | -2.8 | CACNG2 | calcium channel, voltage-dependent, gamma subunit 2 | | 5.8 | |
| SMAD6 | SMAD, mothers against DPP homolog 6 (Drosophila) | -2.8 | CACNA1C | calcium channel, voltage-dependent, L type, alpha 1C subunit | | 4.8 | |
| LEFTY2 | left-right determination factor 2 | -2.8 | JUN | v-jun sarcoma virus 17 oncogene homolog (avian) | | 4.8 | |
| SMAD2 | SMAD, mothers against DPP homolog 2 (Drosophila) | -2.8 | NTRK2 | neurotrophic tyrosine kinase, receptor, type 2 | | 4.7 | |
| SMAD5 | SMAD, mothers against DPP homolog 5 (Drosophila) | -2.6 | MAP2K4 | mitogen-activated protein kinase kinase 4 | | 3.9 | |
| INHBB | inhibin, beta B (activin AB beta polypeptide) | -2.4 | MYC | v-myc myelocytomatosis viral oncogene homolog (avian) | | 3.7 | |
| PPP2CA | protein phosphatase 2 (formerly 2A), catalytic subunit, alpha | -2.4 | CACNA2D3 | calcium channel, voltage-dependent, alpha 2/delta 3 subunit | | 3.6 | |
| TGFB3 | transforming growth factor, beta 3 | -2.3 | RRAS | related RAS viral (r-ras) oncogene homolog | | 3.3 | |
| **CALCIUM SIGNALLING** | |  | RAC1 | ras-related C3 botulinum toxin substrate 1 (rho family, small GTP | | 3.2 | |
| MYLK | myosin, light polypeptide kinase | -6.2 | CACNB4 | calcium channel, voltage-dependent, beta 4 subunit | | 3.2 | |
| EDNRA | endothelin receptor type A | -4.6 | FGF4 | fibroblast growth factor 4 (heparin secretory transforming protein | | 3.2 | |
| PLN | phospholamban | -4.2 | MAPK1 | mitogen-activated protein kinase 1 | | 3.1 | |
| PDGFRB | platelet-derived growth factor receptor, beta polypeptide | -3.3 | MOS | v-mos Moloney murine sarcoma viral oncogene homolog | | 3.1 | |
| AGTR1 | angiotensin II receptor, type 1 | -3.2 | NFKB1 | nuclear factor of kappa light polypeptide gene enhancer in B-cells 1 | | 3 | |
| PPP3CA | protein phosphatase 3 (formerly 2B), catalytic subunit, alpha | -3 | MAPK10 | mitogen-activated protein kinase 10 | | 2.7 | |
| GNAQ | guanine nucleotide binding protein (G protein), q polypeptide | -2.9 | MAP2K5 | mitogen-activated protein kinase kinase 5 | | 2.2 | |
| PDGFRA | platelet-derived growth factor receptor, alpha polypeptide | -2.7 | DUSP8 | dual specificity phosphatase 8 | | 2 | |
| EGFR | epidermal growth factor receptor (erythroblastic leukemia viral | -2.6 | NLK | nemo-like kinase | | 1.9 | |
| BDKRB2 | bradykinin receptor B2 | -2.5 | **TOLL LIKE RECEPTOR** | | |  | |
| ATP2B4 | ATPase, Ca++ transporting, plasma membrane 4 | -2.5 | FOS | v-fos FBJ murine osteosarcoma viral oncogene homolog | | 9 | |
| PPP3R1 | protein phosphatase 3 (formerly 2B), regulatory subunit B, 19kDa, alpha isoform (calcineurin B, type I) | -1.7 | JUN | v-jun sarcoma virus 17 oncogene homolog (avian) | | 6.8 | |
| **MAPK SIGNALING** | |  | MAP2K4 | mitogen-activated protein kinase kinase 4 | | 3.9 | |
| HSPB2 | heat shock 27kDa protein 2 | -5.2 | IFNA8 | interferon, alpha 8 | | 3.7 | |
| DUSP1 | dual specificity phosphatase 1 | -4.6 | RAC1 | ras-related C3 botulinum toxin substrate 1 (rho family, small GTP | | 3.2 | |
| FLNC | filamin C, gamma (actin binding protein 280) | -3.5 | NFKB1 | nuclear factor of kappa light polypeptide gene enhancer in B-cells 1 | | 3.1 | |
| PDGFRB | platelet-derived growth factor receptor, beta polypeptide | -3.3 | CCL3 | chemokine (C-C motif) ligand 3 | | 3 | |
| GNG12 | guanine nucleotide binding protein (G protein), gamma 12 | -3.2 | IFNA13 | interferon, alpha 13 | | 3 | |
| NFATC2 | nuclear factor of activated T-cells, cytoplasmic, calcineurin-dependent 2 | -3.1 | MAPK10 | mitogen-activated protein kinase 10 | | 3.4 | |
| PPP3CA | protein phosphatase 3 (formerly 2B), catalytic subunit, alpha | -3 | CD80 | CD80 molecule | | 2.5 | |
| CASP8 | caspase 8, apoptosis-related cysteine peptidase | -3 | TIRAP | toll-interleukin 1 receptor (TIR) domain containing adaptor protein | | 2.3 | |
| GADD45B | growth arrest and DNA-damage-inducible, beta | -2.8 | **JAK-STAT** | | | | |
| PDGFRA | platelet-derived growth factor receptor, alpha polypeptide | -2.7 | CBLB | Cas-Br-M (murine) ecotropic retroviral transforming sequence b | | | 5.4 |
| AKT3 | v-akt murine thymoma viral oncogene homolog 3 (protein kinase B, gamma) | -2.7 | IL12RB2 | interleukin 12 receptor, beta 2 | | | 4.2 |
| CACNB2 | calcium channel, voltage-dependent, beta 2 subunit | -2.7 | CSF2 | colony stimulating factor 2 (granulocyte-macrophage) | | | 4.2 |
| ZAK | sterile alpha motif and leucine zipper containing kinase AZK | -2.6 | MYC | v-myc myelocytomatosis viral oncogene homolog (avian) | | | 3.7 |
| EGFR | epidermal growth factor receptor (erythroblastic leukemia viral | -2.6 | IFNA8 | interferon, alpha 8 | | | 3.6 |
| NTF3 | neurotrophin 3 | -2.6 | IFNA13 | interferon, alpha 13 | | | 2.9 |
| FGF2 | fibroblast growth factor 2 (basic) | -2.4 | IL2RA | interleukin 2 receptor, alpha | | | 2.9 |
| TGFB3 | transforming growth factor, beta 3 | -2.3 | CRLF2 | cytokine receptor-like factor 2 | | | 2.5 |
| PPP3R1 | protein phosphatase 3 (formerly 2B), regulatory subunit B, 19kDa, alpha isoform (calcineurin B, type I) | -1.7 | PTPN11 | protein tyrosine phosphatase, non-receptor type 11 (Noonan syndrome | | | 2.4 |
| **TOLL LIKE RECEPTOR** | |  | SOCS6 | suppressor of cytokine signaling 6 | | | 2.4 |
| PIK3R1 | phosphoinositide-3-kinase, regulatory subunit 1 (p85 alpha) | -3.4 | CSF2RB | colony stimulating factor 2 receptor, beta, low-affinity | | | 1.3 |
| CCL5 | chemokine (C-C motif) ligand 5 | -3.1 | **WNT- SIGNALLING SYSTEM** | | | | |
| CASP8 | caspase 8, apoptosis-related cysteine peptidase | -3 | JUN | v-jun sarcoma virus 17 oncogene homolog (avian) | | | 4.8 |
| AKT3 | v-akt murine thymoma viral oncogene homolog 3 (protein kinase B, gamma) | -2.7 | WNT4 | wingless-type MMTV integration site family, member 4 | | | 4.2 |
| **JAK-STAT** | |  | WNT8A | wingless-type MMTV member 8A | | | 4.2 |
| PIK3R1 | phosphoinositide-3-kinase, regulatory subunit 1 (p85 alpha) | -3.4 | MYC | v-myc myelocytomatosis viral oncogene homolog (avian) | | | 3.7 |
| LEPR | leptin receptor | -3.2 | FZD2 | frizzled homolog 2(Drosophila) | | | 3.6 |
| PRLR | prolactin receptor | -3.2 | RAC1 | ras-related C3 botulinum toxin substrate 1 (rho family, small GTP | | | 3.2 |
| SOCS2 | suppressor of cytokine signaling 2 | -3.1 | PPARD | peroxisome proliferative activated receptor, delta | | | 3.1 |
| CCND2 | cyclin D2 | -2.9 | GSK3B | glycogen synthase kinase 3 beta | | | 2.8 |
| JAK2 | Janus kinase 2 (a protein tyrosine kinase) | -2.9 | MAPK10 | mitogen-activated protein kinase 10 | | | 2.7 |
| AKT3 | v-akt murine thymoma viral oncogene homolog 3 (protein kinase B, gamma) | -2.7 | CTBP1 | C-terminal binding protein 1 | | | 2.7 |
| **PHOSPHATIDYLINOSITOL SIGNALING SYSTEM** | | | CAMK2G | calcium/calmodulin-dependent protein kinase (CaM kinase) II gamma | | | 2.3 |
| INPP5A | inositol polyphosphate-5-phosphatase, 40kDa | -3.4 | CAMK2D | calcium/calmodulin-dependent protein kinase (CaM kinase) II delta | | | 2.1 |
| PIK3R1 | phosphoinositide-3-kinase, regulatory subunit 1 (p85 alpha) | -3.4 | NLK | nemo-like kinase | | | 1.9 |
| PTEN | phosphatase and tensin homolog (mutated in multiple advanced cancers | -2.8 | CTNNB1 | catenin (cadherin-associated protein), beta 1, 88kDa | | | 1.7 |
| PIK3C2A | phosphoinositide-3-kinase, class 2, alpha polypeptide | -2.7 | **CELL CYCLE** | | | |  |
| **WNT- SIGNALLING SYSTEM** | | | MYC | v-myc myelocytomatosis viral oncogene homolog (avian) | | | 3.7 |
| SFRP4 | secreted frizzled-related protein 4 | -5.4 | CDK6 | cyclin-dependent kinase 6 | | | 3.2 |
| DAAM2 | dishevelled associated activator of morphogenesis 2 | -4.5 | CHEK2 | CHK2 checkpoint homolog (S. pombe) | | | 3 |
| PRICKLE1 | prickle-like 1 (Drosophila) | -3.9 | BUB1 | BUB1 budding uninhibited by benzimidazoles. | | | 2.9 |
| FBXW11 | F-box and WD-40 domain protein 11 | -3.5 | GSK3B | glycogen synthase kinase 3 beta | | | 2.8 |
| DAAM1 | dishevelled associated activator of morphogenesis 1 | -3.4 | ANAPC10 | anaphase promoting complex subunit 10 | | | 2.6 |
| NFATC2 | nuclear factor of activated T-cells, cytoplasmic, calcineurin-dependent 2 | -3.1 | CDC25C | cell division cycle 25C | | | 2.5 |
| PPP3CA | protein phosphatase 3 (formerly 2B), catalytic subunit, alpha | -3 | CDKN2A | cyclin-dependent kinase inhibitor 2A (melanoma, p16, inhibits CDK4) | | | 1.5 |
| CCND2 | cyclin D2 | -2.9 |  |  | | |  |
| FZD7 | frizzled homolog 7 (Drosophila) | -2.9 |  |  | | |  |
| SMAD2 | SMAD, mothers against DPP homolog 2 (Drosophila) | -2.8 |  |  | | |  |
| PPP2R2A | protein phosphatase 2 (formerly 2A), regulatory subunit B (PR 52), alpha isoform | -2.8 |  |  | | |  |
| CSNK1A1 | casein kinase 1, alpha 1 | -2.7 |  |  | | |  |
| PPP2CA | protein phosphatase 2 (formerly 2A), catalytic subunit, alpha | -2.4 |  |  | | |  |
| PPP3R1 | protein phosphatase 3 (formerly 2B), regulatory subunit B, 19kDa, alpha isoform (calcineurin B, type I) | -1.7 |  |  | | |  |
| **CELL CYCLE** | |  |  |  | | |  |
| CDKN1C | cyclin-dependent kinase inhibitor 1C (p57, Kip2) | -3.7 |  |  | | |  |
| CCND2 | cyclin D2 | -2.9 |  |  | | |  |
| SMAD2 | SMAD, mothers against DPP homolog 2 (Drosophila) | -2.8 |  |  | | |  |
| GADD45B | growth arrest and DNA-damage-inducible, beta | -2.8 |  |  | | |  |
| ABL1 | v-abl Abelson murine leukemia viral oncogene homolog 1 | -2.5 |  |  | | |  |
| TGFB3 | transforming growth factor, beta 3 | -2.3 |  |  | | |  |

Each pathway contains down or up modulated genes in cervical carcinoma that were SAM significantly selected. Hence, left column corresponds to down modulated genes, whereas right column corresponds to up regulated.

^a^Ratio is expressed as the Log2 of expression values in tumour samples versus normal samples for each gene
